# Development of an SNP-based high-density linkage map and QTL analysis for bruchid (*Callosobruchus maculatus* F.) resistance in black gram (*Vigna mungo* (L.) Hepper)

**DOI:** 10.1038/s41598-019-40669-5

**Published:** 2019-03-08

**Authors:** Prakit Somta, Jingbin Chen, Chutintorn Yundaeng, Xingxing Yuan, Tarika Yimram, Norihiko Tomooka, Xin Chen

**Affiliations:** 1 Institute of Industrial Crops, Jiangsu Academy of Agricultural Sciences, 50 Zhongling Street, Nanjing, 210014 China; 2 Department of Agronomy, Faculty of Agriculture at Kamphaeng Saen, Kasetsart University, Kamphaeng Saen Campus, Kamphaeng Saen, Nakhon Pathom 73140 Thailand; 3 Center for Agricultural Biotechnology (AG-BIO/PEDRO-CHE), Kasetsart University, Kamphaeng Saen Campus, Kamphaeng Saen, Nakhon Pathom 73140 Thailand; 4 Genetic Resources Center, Gene Bank, National Agriculture and Food Research Organization, 2-1-2 Kannondai, Tsukuba, Ibaraki 305-8602 Japan

## Abstract

Black gram (*Vigna mungo* var. *mungo*) is an important pulse crop in Asia. The cowpea weevil (*Callosobruchus maculatus*) is a stored-seed insect pest (seed weevil/bruchid) that causes serious postharvest losses in pulse crops, including black gram. In this study, we constructed a high-density linkage map for black gram and identified quantitative trait loci (QTLs) for *C*. *maculatus* resistance. A recombinant inbred line (RIL) population of 150 lines from a cross between BC48 [cultivated black gram (var. *mungo*); bruchid-susceptible] and TC2210 [wild black gram (var. *silvestris*); bruchid-resistant] were used to construct a linkage map of 3,675 SNP markers from specific-locus amplified fragment sequencing. The map comprised 11 linkage groups spanning 1,588.7 cM with an average distance between adjacent markers of 0.57 cM. Seeds of the RIL population grown in 2016 and 2017 were evaluated for *C*. *maculatus* resistance through two traits; the percentage of damaged seeds (PDS) and infestation severity progress (AUDPS). Inclusive composite interval mapping identified three QTLs each for PDS and AUDPS. Two QTLs, *qVmunBr6*.1 and *qVmunBr6*.2, mapped about 10 cM apart on linkage group 6 were common between PDS and AUDPS. Comparative genome analysis revealed that *qVmunBr6*.*1* and *qVmunBr6*.*2* are new loci for *C*. *maculatus* resistance in *Vigna* species and that genes encoding a lectin receptor kinase and chitinase are candidates for *qVmunBr6*.*2*. The high-density linkage map constructed and QTLs for bruchid resistance identified in this study will be useful for molecular breeding of black gram.

## Introduction

Black gram (*Vigna mungo* (L.) Hepper) is an important legume crop of Asia. It is cultivated as a component of various cropping systems that cover over four million hectares, principally in India, Myanmar, Pakistan, Bangladesh and Thailand. In South Asia, dry black gram seeds are mainly consumed as a thick soup, while seed flour is used to prepare several dishes. In Thailand and Japan, black gram seeds are popular in the sprout industry. Nonetheless, the average seed yield of blackgram is very low, e.g. about 650–800 kg/ha in Thailand and India. In addition, after harvest, black gram seeds are frequently destroyed by bruchids (seed weevils) (Coleoptera: Bruchidae). Generally, the seeds of all *Vigna* crops are attacked by bruchids. The two most important and damaging bruchid species for *Vigna* crops are the cowpea weevil (*Callosobruchus maculatus* L.) and azuki bean weevil (*Callosobruchus chinensis* F.)^1^. These two bruchid species are widely distributed across nearly all continents due to international seed trading. Bruchid infestation of legume crops initially occurs in the field where female bruchids lay eggs on young pods and larvae bore through the pods into the seeds, in which they grow and develop into adults by consuming seed nutrients. The infestation starts in the field and continues during storage. After harvest, the adult bruchids emerge from the seeds and start a new infestation by laying eggs directly on seeds, which can result in the total loss of a seed lot within 2–4 months^1^. Chemical fumigation is mostly used to control the bruchids, but it is not practical for small-scale farmers and traders and is unsafe for human health and the environment. The most practical and sustainable method for managing bruchid infestation is the use of resistant cultivars.

Sources of bruchid resistance have been reported for some *Vigna* crops, although resistant germplasm is rare and in some cases not highly effective^2–7^ due to the complex interaction between resistance and insect species/biotypes. Cultivated black gram (*V*. *mungo* var. *mungo*) is completely resistant to *C*. *chinensis*, but highly susceptible to *C*. *maculatus*^6,7^. In contrast, its wild progenitor, *Vigna mungo* var. *silvestris* Lukoki, Maréchal & Otoul, is resistant to both of these bruchid species^6,7^. Compared with cultivated black gram, wild black gram shows a lower percentage of adult emergence (PAE), lower percentage of damaged seeds (PDS), and longer developmental period (DP) for *C*. *maculatus*^8^. Its resistance is due to antibiosis of seeds^9^. Based on PAE evaluation, it was reported that the resistance to *C*. *maculatus* in *V*. *mungo* var. *silvestris* is controlled by two dominant duplicate genes, designated *Cmr1* and *Cmr2*^6^. Quantitative trait loci (QTLs) for the resistance were located on a linkage map of a recombinant inbred line (RIL) population (*V*. *mungo* var. *silvestris* × *V*. *mungo* var. *mungo*) using 428 markers (381 dominant and 47 co-dominant markers)^9^. Two QTLs for PAE, *Cmrae1*.*1* and *Cmrae1*.*2*, were identified on linkage groups (LGs) 3 and 4, respectively, while six QTLs were identified for DP; two (*Cmrdp1*.*1* and *Cmrdp1*.*2*) on LG1, three (*Cmrdp1*.*1*, *Cmrdp1*.*1* and *Cmrdp1*.*1*) on LG2, and one (*Cmrdp1*.*1*) on LG10^9^. Nonetheless, none of these QTLs have been used in genetic improvement of the traits because of a lack of closely linked markers.

Molecular breeding by marker-assisted selection relies on DNA markers closely linked to the trait of interest^10^. Thus, in marker-assisted selection, high-resolution mapping of a trait should be conducted to identify markers closely linked to the target trait. High-resolution mapping is also useful to identify the gene or candidate gene underlying the trait. Currently, advanced sequencing technologies enable scientists to directly detect thousands of single nucleotide polymorphisms (SNPs) and insertions and deletions (indels) in organisms rapidly at low cost. Several sequencing-based genotyping methods have been developed such as genotyping-by-sequencing (GBS)^11^, restriction-associated DNA sequencing (RAD-seq)^12^ (Baird *et al*. 2008), diversity array technology sequencing (DArTseq)^13^ and specific-locus amplified fragment sequencing (SLAF-seq)^14^.

In this paper we report the development of a high-density linkage map for black gram using the SLAF-seq technique and the identification of QTLs controlling seed resistance to *C*. *maculatus*.

## Results

### SLAF sequencing data and genotyping

After preprocessing, 79.49 Gb of raw data containing 399.60 M reads were generated. On average, the Q30 (quality score of at least 30, indicating a 1% chance of error, and thus 99% confidence) was 93.02% and the GC content was 39.76%. The numbers of reads for TC2210 and BC48 were 12,553,056 and 13,266,999, respectively. The read numbers for each F_2_ individual ranged from 1,178,049 to 2,363,336 with an average of 2,491,876. After read clustering, a total of 577,828 SLAFs were detected, and the average sequencing depth was 35.65-fold, 26.00-fold and 9.28-fold for BC48, TC2210 and the progeny, respectively.

From the 577,828 SLAFs, after filtering SLAF markers lacking parent information and showing low read depth, 30,978 polymorphic markers were successfully genotyped and grouped into eight segregation patterns viz., ab × cd, ef × eg, hk × hk, lm × ll, nn × np, aa × bb, ab × cc, and cc × ab. In total, there were 8,913 markers showing the segregation pattern aa × bb.

### Features of the genetic map

**Figure 1:**
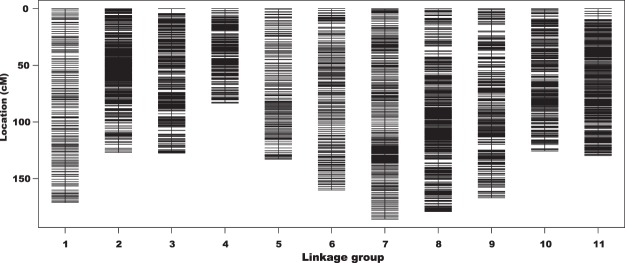
High-density linkage map of black gram (*Vigna mungo*) constructed from a black gram RIL population derived from the cross BC48 (cultivated) × TC2210 (wild). The map is composed of 3,675 SNP markers generated from specific-locus amplified fragment sequencing.

After careful screening, 4,588 markers were used for genetic linkage analysis, among which 3,675 (80.1%) were clustered into a linkage map of 11 LGs (Fig. 1). Table 1 summarizes the characteristics of the linkage map for black gram constructed in this study. The map had a total length of 1,588.7 cM with an average distance between adjacent markers of 0.57 cM. The number of markers per LG ranged from 134 on LG10 to 665 on LG3 with an average of 334.1 markers. LG4 was the shortest LG (83.4 cM), while LG7 was the longest (185.8 cM) (Fig. 1). The average length of the LGs was 144.43 cM. The marker density was 2.3 markers per cM.

**Table 1:** Characteristics of the high-density linkage map of black gram (*Vigna mungo*) constructed from an F_9_ RIL population derived from a cross between BC48 and TC2210 using SNP-based SLAF-seq markers.

Linkage group	Length (cM)	No. of SNP markers	Average distance between adjacent markers (cM)
LG1	171.1	134	1.29
LG2	126.9	432	0.29
LG3	127.5	307	0.42
LG4	83.4	229	0.37
LG5	132.8	136	0.98
LG6	160.0	185	0.87
LG7	185.8	239	0.78
LG8	179.1	532	0.34
LG9	166.9	413	0.41
LG10	125.6	403	0.31
LG11	129.6	665	0.20
Total	1588.7	3,675	—
Average	144.4	334.1	0.57

### *C*. *maculatus* resistance in the parents and the RIL population

**Figure 2:**
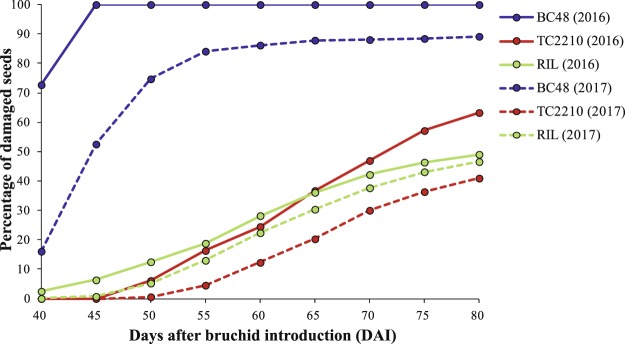
Pattern of seed infestation by *Callosobruchus maculatus* in BC48 (cultivated), TC2210 (wild) and a RIL population of the cross BC48 × TC2210 in two years (2016 and 2017).

The pattern of *C*. *maculatus* infestation in BC48, TC2210 and the RIL population is shown in Figure 2. BC48 and TC2210 were contrasting in both PDS and AUDPS caused by *C*. *maculatus*, while the RIL population showed a similar infestation pattern to TC2210. When BC48 and TC2210 and the RIL population were grown in 2016, the PDS of BC48 and TC2210 was 100% and 63.27%, respectively. The PDS in the RIL population ranged from 2.0% to 100% with a mean of 52.82%. AUDPS was calculated to indicate the progression of *C*. *maculatus* infestation severity. The AUDPS of BC48 and TC2210 was 521.82 and 163.67, respectively, indicating that TC2210 was damaged slower than BC48. The AUDPS in the RIL population ranged from 3.11 to 469.60 with a mean of 158.

In 2017, the PDS of BC48 was 40.96% and that of TC2210 was 89.19%. PDS in the RIL population ranged from 10.15% to 81.63% with a mean of 47.26%. The AUDPS of BC48 was 314.25 and that of TC2210 was 96.72. The AUDPS in the RIL population varied from 20.21 to 273.21 with a mean of 47.26.

When the data from the two years were combined, the PDS of BC48 and TC2210 was 94.59% and 52.11%, respectively. PDS in the RIL population varied between 7.67% and 89.60% with a mean of 50.09%. The AUDPS of BC48 and TC2210 was 418.03 and 130.19, respectively. The AUDPS in the population ranged from 15.03 to 369.12 with a mean of 141.11.

**Figure 3:**
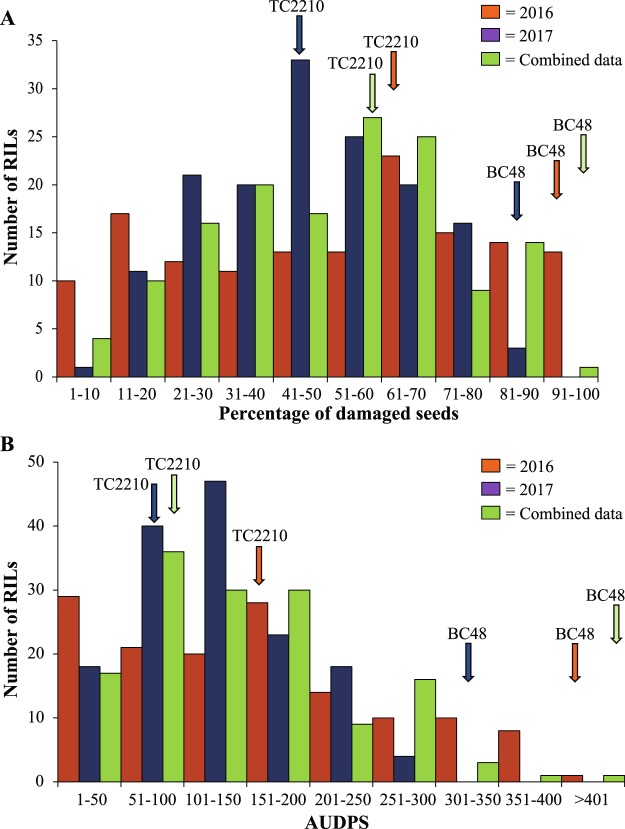
Percentage of damaged seeds (**A**) and infestation severity progress as indicated by area under disease progress stair (AUDPS) (**B**) caused by *Callosobruchus maculatus* in a black gram RIL population of the cross BC48 × TC2210 in two years (2016 and 2017).

The frequency distribution of PDS and AUDPS in the RIL population in 2016, 2017 and both years combined showed a continuous distribution (Fig. 3). In all cases, both PDS and AUDPS showed transgressive segregation. Many RILs had lower PDS and AUDPS values than the resistant parent TC2210.

### Heritability and QTLs for *C*. *maculatus* resistance

The narrow-sense heritability estimated for PDS and AUDPS caused by *C*. *maculatus* in the F_10_ RIL population was relatively low, being 38.58% and 41.93, respectively.

**Figure 4:**
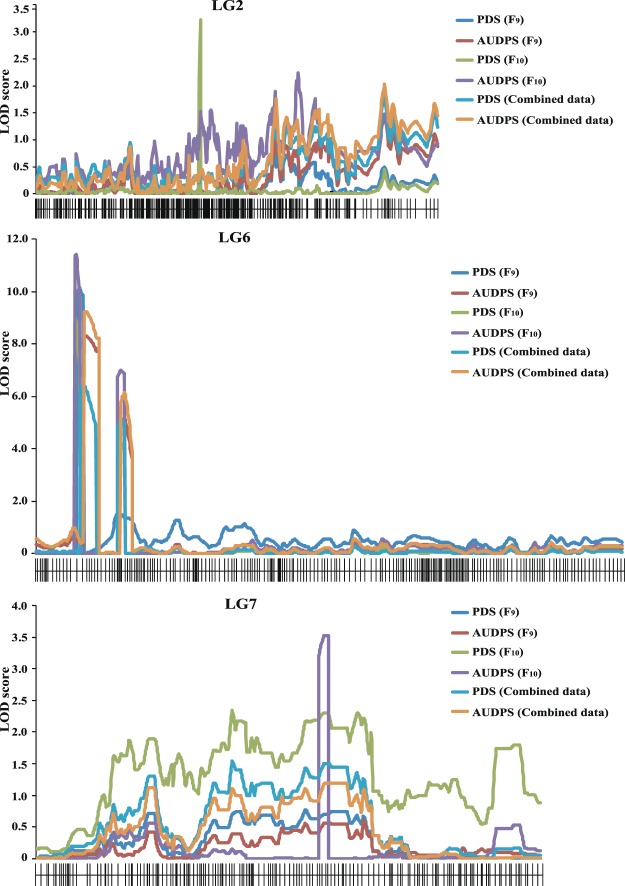
LOD graphs of the QTLs identified for the percentage of damaged seeds and area under disease progress stair (AUDPS) caused by *Callosobruchus maculatus* in a black gram RIL population of the cross BC48 × TC2210 in two years (2016 and 2017).

Inclusive composite interval mapping (ICIM) was conducted in the RIL population to locate QTLs for *C*. *maculatus* resistance on the linkage map. Table 2 summarizes the QTLs detected for resistance. Figure 4 shows a LOD graph of the detected QTLs on the linkage maps. In total, six QTLs were detected for the two traits related to resistance; three for PDS and three for AUDPS. At all the detected QTLs, allele(s) from TC2011 increased resistance by reducing PDS and/or AUDPS.

**Table 2:** QTLs identified for seed resistance to *Callosobruchus maculatus* in a RIL population of the cross between cultivated black gram ‘BC48’ and wild black gram ‘TC2210’ by inclusive composite interval mapping.

Trait^a^	LG^b^	QTL name	2016 (F_9_)				2017 (F_10_)				Combined			
			Position^c^	LOD score	PVE^d^ (%)	Add^e^	Position^c^	LOD score	PVE^d^ (%)	Add^e^	Position^c^	LOD score	PVE^d^ (%)	Add^e^
PDS	2	*qCm_PDS2*.*1*	Not found				51.8	3.22	7.95	−4.43	Not found			
	6	*qCm_PDS6*.*1*	11.6	10.14	28.06	−15.77	10.4	9.07	24.32	−8.00	12.8	6.43	22.40	−8.23
	6	*qCm_PDS6*.*2*	Not found				23.0	4.23	10.61	−5.58	23.0	5.10	17.36	−7.60
AUDPS	6	*qCm_AUDPS6*.*1*	13.0	8.33	28.76	−47.05	10.8	11.43	26.75	−28.83	13.0	9.25	30.04	−35.94
	6	*qCm_AUDPS6*.*2*	24.0	5.20	17.37	−38.50	23.0	7.00	15.26	−22.91	24.0	6.16	19.60	−30.56
	7	*qCm_AUDPS7*.*1*	Not found				107.40	3.53	7.28	−14.50	Not found			

^a^PDS = percentage of damaged seeds, AUDPS = area under the disease progress stair.

^b^linkage group.

^c^Position (centimorgans) on the linkage map.

^d^Phenotypic variance explained by the QTL.

^e^Additive effect.

In 2016, ICIM identified one QTL for PDS (*qCm_PDS6*.*1*) and two QTLs for AUDPS (*qCm_AUDPS6*.*1* and *qCm_AUDPS6*.*2*). All of these QTLs were on LG6. *qCm_PDS6*.*1* and *qCm_AUDPS6*.*1* were located very near to each other (about 1 cM apart) on LG6 and each explained about 28% of the total variation of each trait in the RIL population. *qCm_AUDPS6*.*2* was about 12 cM away from *qCm_PDS6*.*1* and *qCm_AUDPS6*.*1*. It accounted for 17.37% of the total variation of AUDPS in the RIL population.

In 2017, six QTLs for resistance were detected; three for PDS and three for AUDPS. Of the six QTLs, three (*qCm_PDS6*.*1 qCm_AUDPS6*.*1* and *qCm_AUDPS6*.*2*) were also found in 2016, while the other three (*qCm_PDS2*.*1* and *qCm_PDS6*.*2* for PDS and *qCm_AUDPS7*.*1* for AUDPS) were found only in 2017. *qCm_PDS6*.*1*, *qCm_AUDPS6*.*1* and *qCm_AUDPS6*.*2*, which were detected in 2017, showed similar percentages of variance explained (PVEs) to those in 2016. *qCm_PDS2*.1, *qCm_PDS6*.*2*, and *qCm_AUDPS7*.*1* were on LGs 2, 6 and 7, respectively. *qCm_PDS2*.*1* and *qCm_AUDPS7*.*1* had PVEs of 7.95% and 7.28%, respectively, while *qCm_PDS6*.*2* had a PVE of about 15%.

In the case of the combined data, four QTLs were detected for resistance; two for PDS (*qCm_PDS6*.*1* and *qCm_PDS6*.*2* and two for AUDPS (*qCm_AUDPS6*.*1* and *qCm_AUDPS6*.*2*). These QTLs were mapped to the same or very similar positions to those in the years 2016 and/or 2017. They accounted for 22.40%, 17.36%, 30.04%, and 19.60% of the trait variation in the combined data.

## Discussion

Although black gram is an important legume crop in Asia, genetics and breeding of black gram, especially molecular breeding, has lagged behind other legumes with similar levels of importance. Only two genetic linkage maps have been previously developed for black gram. In addition, only a few gene mapping studies have been conducted. In this study, we successfully constructed a high-density genetic linkage map and located QTLs for bruchid resistance on the linkage map.

### High-density linkage map of black gram

The linkage map constructed in this study is the first high-density linkage map developed for black gram. The first linkage map developed for black gram comprised only 148 marker loci (59 RFLP, 61 SSR, 27 AFLP and 1 morphological markers)^15^, while the second map comprised 428 marker loci but most of the markers were dominant (254 AFLP, 86 RAPD, 41 ISSR and 47 SSR markers)^16^. The high-density linkage map developed here comprised 3,675 SNP-based SLAF-seq markers that resolve the 11 haploid chromosomes of black gram (Fig. 1 and Table 1). Therefore, the map is useful for gene mapping of agronomically important traits in black gram.

### Heritability for *C*. *maculatus* resistance

The narrow-sense heritability estimated for *C*. *maculatus* resistance in black gram in this study was only about 40%. This indicated that resistance is principally affected by the environment. This heritability value in this study is very low compared with the value previously estimated for *C*. *maculatus* resistance by^9^, who reported up to 99% heritability for resistance in black gram. The contrasting results between our study and that of ^9^ may stem from (i) the difference in the sources of resistance and/or (ii) differences in biotypes (strains) of *C*. *maculatus*. Additional study is necessary to explain the contrasting results.

### QTLs for *C*. *maculatus* resistance in black gram

In total, six QTLs were identified for two traits related to *C*. *maculatus* resistance in the wild black gram TC2210 (Table 2). However, because *qCm_PDS6*.*1*, *qCm_PDS6*.*2*, *qCm_AUDPS6*.*1* and *qCm_AUDPS6*.*2* were consistently identified in 2016, 2017 and the combined data, and *qCm_PDS6*.*1* and *qCm_AUDPS6*.*1*, and *qCm_PDS6*.*2* and *qCm_AUDPS6*.*2* were mapped to the same or very similar locations, we considered *qCm_PDS6*.*1* and *qCm_AUDPS6*.*1* to be the same QTL and named it *qVmunBr6*.*1* and *qCm_PDS6*.*2* and *qCm_AUDPS6*.*2* to be the same QTL and named it *qVmunBr6*.*2*. Therefore, four QTLs were associated with *C*. *maculatus* resistance in the wild black gram TC2210. Nonetheless, *qCm_PDS2*.*1* and *qCm_AUDPS7*.*1* were found only in one environment and were not found in the combined data. They showed lower PVEs of about 7–8%. These two QTLs can be considered modifying loci for resistance. As a result, only two QTLs, *qVmunBr6*.*1* and *qVmunBr6*.*2*, were confirmed for the *C*. *maculatus* resistance in TC2210. Based on Mendelian segregation analysis of damaged seeds^6^, found that *C*. *maculatus* resistance in the wild black gram accession “Trombay wild” was controlled by two duplicate loci that were designated *Cmr*_*1*_ and *Cmr*_*2*_. This finding more or less agrees with our result that *C*. *maculatus* resistance in the wild black gram TC2210 is controlled by two major loci, *qVmunBr6*.*1* and *qVmunBr6*.*2*, in combination with one or two modifying loci.^9^ identified as many as eight QTLs for *C*. *maculatus* resistance in black gram; two for adult emergence (*Cmrae*1.1 and *Cmrae*1.2) and six for developmental period. The QTLs *Cmrae*1.1 and *Cmrae*1.2 were mapped to different LGs. As a result, these QTLs appear to be different from the QTLs *qVmunBr6*.*1* and *qVmunBr6*.*2* for *C*. *maculatus* resistance in TC2210, which were mapped to the same LG (Table 2). Nonetheless, the contrasting findings suggest that the wild black gram used in this study and that used in^9^ possess different genetic bases for resistance. Further investigation is necessary to clarify the genetics of resistance in different wild black gram germplasm.

Although the transgressive segregation in PDS and AUDPS in the RIL population (Fig. 3) suggested that both TC2210 and BC48 possessed resistance and susceptible genes, alleles from TC2210 conferred resistance at all QTLs detected for the resistance. This suggests that some resistance QTLs contributed by BC48, if any exist, were not detected in this study. Such QTLs may have small genetic effects or be located in genome regions where the linkage map did not have much coverage.

Molecular mapping of *C*. *maculatus* resistance has been reported in other *Vigna* species including mungbean^17,18^, rice bean^19,20^ and wild azuki bean (*Vigna nepalensis*)^21^. We compared the major QTLs for *C*. *maculatus* identified in these studies and our study (*qVmunBr6*.*1* and *qVmunBr6*.*2*) by conducting BLASTN analysis of the SSR marker sequences linked to the QTLs against the reference genomes of mungbean^22^ and azuki bean^23^. We found that the chromosome locations of *qVmunBr6*.*1* and *qVmunBr6*.*2* were chromosome 8 of mungbean and chromosome 8 of azuki bean, and were different from the chromosome locations of the bruchid resistance QTLs in other *Vigna* species (Supplementary Table S1). This indicates that the genes controlling *C*. *maculatus* resistance in black gram are different from those in other species and also suggests that there are diverse mechanisms of resistance to *C*. *maculatus* in *Vigna* species. These diverse mechanisms of resistance will be useful for sustainable breeding to manage *C*. *maculatus* in *Vigna* species.

### Candidate genes for *C*. *maculatus* resistance in black gram

Because a reference genome sequence of black gram was not available and there is high genome conservation between species in the genus *Vigna*^22^, we identified candidate genes for bruchid resistance in black gram using the genome sequences of mungbean^22^, azuki bean^23^ and cowpea^24^. The BLASTN search revealed that the markers around *qVmunBr6*.*1* corresponded to two or three chromosomes in these *Vigna* species (Supplementary Table S2). Therefore, we were not able to identify the candidate genes for resistance in *qVmunBr6*.*1*. Nonetheless, the search revealed that the markers Marker14881, Marker9514 and Marker15884 flanking *qVmunBr6*.*2* corresponded to a 160.2 kb region of mungbean chromosome 8 (Chr08: 39,937,678..40,097,824), a 173.7 kb region of chromosome 8 of azuki bean (Chr08: 7,100,761..7,274,445) and a 150.9 kb region of cowpea chromosome 7 (Chr07: 34,054,059.. 34204986) (Supplementary Table S2). We scanned the annotated genes in these regions and found that *LOC106770636* and *Vigun07g218900* encoding lectin receptor kinase (LecRK) were present in the 160.2 kb and 150.9 kb regions of mungbean and cowpea, respectively. The seed lectins of some legume species have been shown to be highly toxic to *C*. *maculatus*^25–27^. Because LecRK possesses a lectin domain, it may be toxic to *C*. *maculatus*. In addition, we found that the genes *LOC106771180* (Chr08: 40114473..40115564), *Vigan*.*08G086800*.*01* (Chr08: 7,093,766..7,095,438) and *Vigun07g219300* (Chr07: Chr07: 34,212,181..34,213,351) encoding chitinase were located very close to the 160.2 kb region of mungbean, 173.7 kb region of azuki bean and 150.9 kb region of cowpea, respectively. A chitinase isolated from cowpea seeds was shown to affect the development of *C*. *maculatus* larvae, although it did not much affect their survival^28^. Chitinase is among the seed chemicals that are associated with resistance to *C*. *chinensis* in mungbean^29^. Recently, soybean seed coat chitinase was shown to be highly toxic to *C*. *maculatus*^30^. Thus, the genes for LecRK and chitinase can be considered candidate genes at the QTL *qVmunBr6*.*2* for *C*. *maculatus* resistance in black gram.

## Materials and Methods

### Plant materials and DNA extraction

A RIL population of 150 lines developed by a single-seed descent method from the cross ‘BC48’ × ‘TC2210’ was used in this study. BC48 is a cultivated blackgram from Thailand and is susceptible to *C*. *maculatus*, while TC2210 is a wild blackgram from India and is resistant to *C*. *maculatus*^7^. TC2210 showed a lower percentage of damaged seeds and slower progress of seed damage caused by bruchids compared with BC48.

In 2016, the F_9_ RILs (one plant per line) and their parents were grown under field conditions using 0.5 × 0.5 m spacing at Kasetsart University, Kamphaeng Sean Campus (KU-KPS), Nakhon Pathom, Thailand during October to December. At maturity, the pods of each plants were harvested separately for bruchid evaluation. Genomic DNA was extracted from young leaves of the F_9_ RILs and parents using a CTAB method^31^.

In 2017, the F_10_ RILs and the parents were grown in a randomized complete block design with two replicates under field conditions at the KU-KPS during June to August. The spacing between rows was 0.5 m and the spacing between plants in the same row was 0.25 m. In each replicate, each entry comprised 10 plants, five of which were randomly selected and harvested. The seeds harvested from each individual plant were used for bruchid resistance evaluation.

### SLAF-seq analysis

Genomic DNA extracted from the parents and 150 RILs was used for SLAF library construction and sequencing as per^14^ with minor modifications. Briefly, a pilot experiment for SLAF analysis was performed using the reference genome of mungbean^22^. Based on the results of the pilot experiment, a SLAF library was prepared. Genomic DNA from each entry was digested at 37 °C with *Rsa*I and *Hae*III (NEB, Ipswich, MA, USA), incubated with the Klenow fragment (3′ → 5′exonuclease) (NEB) and dATP at 37 °C to add a single-nucleotide A overhang to the digested fragments, and the A-tailed DNA fragments were then ligated to Duplex Tag-labelled sequencing adapters (PAGE purified, Life Technologies) using T4 DNA ligase. PCR was performed using diluted restriction-ligation DNA samples, dNTPs, Q5® High-Fidelity DNA Polymerase and PCR primers (forward primer: 5′-AATGATACGGCGACCACCGA-3′, reverse primer: 5′-CAAGCAGAAGACGGCATACG-3′) (PAGE-purified, Life Technologies). The PCR products were then purified using Agencourt AMPure XP beads (Beckman Coulter, High Wycombe, UK) and pooled. The pooled samples were separated by 2% agarose gel electrophoresis. Fragments ranging from 314 to 414 bp (with indexes and adaptors) in size were excised and purified using a QIAquick gel extraction kit (Qiagen, Hilden, Germany). The gel-purified products were diluted. Pair-end sequencing (each end 125 bp) was performed using an Illumina HiSeq 2500 system (Illumina, Inc; San Diego, CA, USA) according to the manufacturer’s recommendations at the Biomarker Technologies Corporation (Beijing, China).

### Sequence data grouping and genotyping

The reads generated from sequencing were compared with the mungbean reference genome sequence^22^ using BWA. SLAF marker identification and genotyping were performed as per^14^. Low-quality reads (quality score < 20e) were eliminated and the raw reads were assigned to 150 F_2_ individual samples according to the duplex barcode sequences. After trimming each high-quality read, the clean reads were clustered together according to their sequence identities. Sequences mapping to the same locus with over 90% identity were defined as one SLAF locus. SNP loci between the two parents were detected and SLAFs with >3 SNPs were removed. The alleles of each SLAF were defined according to the parental reads with a sequence depth >6-fold and offspring reads with a sequence depth >2-fold. In black gram, which is a diploid species, one locus contains a maximum of four genotypes; thus, SLAF loci with >4 alleles were discarded and SLAFs with 2, or 3, or 4 alleles were considered potential markers. Polymorphic markers were grouped into eight segregation patterns^14^, but only markers showing the segregation pattern aa × bb were selected. To ensure the genotyping quality, genotype scoring was conducted using a Bayesian approach as described by^14^. Then, three processes were performed to screen for high-quality markers; (i) markers with average sequence depths <6-fold in the parents and <1-fold in the progeny were discarded, (ii) markers with >40% missing data were removed, and (ii) markers with significant segregation distortion (*P* < 0.01) were initially excluded from the genetic map construction and then added later as accessory markers.

### Genetic map construction

Polymorphic SLAF markers were partitioned primarily into LGs based on their locations on the mungbean reference genome. Next, modified logarithm of odds (MLOD) scores between markers were calculated to further confirm the robustness of the markers for each LG. Markers with MLOD scores <6 were filtered prior to ordering. The HighMap strategy^32^ was utilized to order the SLAF markers and correct genotyping errors within LGs. Firstly, recombinant frequencies and LOD scores were calculated by two-point analysis to infer linkage phases. Then, enhanced Gibbs sampling, spatial sampling and simulated annealing algorithms were combined to conduct an iterative process of marker ordering^33,34^. The error correction strategy of SMOOTH^35^ was implemented according to the parental contribution of genotypes, and a *k*-nearest neighbour algorithm was applied to impute missing genotypes^36^. Skewed markers were added into this map by applying a multi-point maximum likelihood method. Map distances were calculated using Kosambi’s mapping function^37^.

### Evaluation of seed resistance to *C*. *maculatus*

A culture of *C*. *maculatus* was reared on seeds of the susceptible mungbean cultivar ‘Kamphaeng Saen 1’ and kept in a room at 30 °C and 50% relative humidity. Evaluation of *C*. *maculatus* resistance was conducted as per^38^ with minor modifications. The seeds harvested from each plant were subjected to resistance evaluation. Forty seeds from each plant were put in a plastic box. Then, 10 pairs (10 males and 10 females) of newly emerged *C*. *maculatus* adults were introduced into the box for egg laying for 7 days and then removed. The infested seeds were maintained at 30 °C and 50% relative humidity. The numbers of seeds damaged by the bruchids were recorded at 40 days after bruchid introduction (DAI), and then at every 5 days until 80 DAI. Each time the seeds were assessed, the damaged seeds were removed from the boxes. Seeds of both parents were also included in the resistance evaluation. The cumulative number of total seeds damaged by the bruchid (seeds with holes) at each counting date was calculated and converted into a percentage. Then, the percentage of damaged seeds was used to calculate the area under disease progress stair (AUDPS)^39^. AUDPS is an improved version of the area under disease progress curve (AUDPC), which indicates progression of disease severity in plants. In this study, AUDPS was used as an indicator for the progression of bruchid infestation severity. The percentage of damaged seeds at 80 DAI and the AUDPS value of each RIL were used for data analysis.

### Heritability estimation for resistance

The narrow-sense heritability (*h*^2^) of PDS and AUDPS was determined for the RIL population grown in the replicated experiment (F_10_). Analysis of variance of PDS and AUDPS was conducted, and then the *h*^2^ of each trait was calculated using the following formula:

$${h}^{2}={\sigma }_{{\rm{g}}}^{2}/[{\sigma }_{{\rm{g}}}^{2}+({\sigma }_{e}^{2}/r)],$$

where ${\sigma }_{{\rm{g}}}^{2}\,{\rm{and}}\,{\sigma }_{e}^{2}$ are the variances of the RILs and experimental error, respectively, and *r* is the number of replicates.

### QTL analysis

Because several SLAF-seq markers were often mapped to the same position, in such cases only one marker was selected and used for QTL analysis. QTL analysis was conducted using the ICIM method^40^ implemented in the QTL IciMapping 4.1 software^41^. The PDS and AUDPS were used to locate QTLs for resistance. ICIM was performed every 0.2 cM with a probability in stepwise regression (PIN) of 0.001. The significant LOD threshold for QTLs of each trait was determined with a 3,000-permutation test at *P* = 0.05.

## Supplementary information

Supplementary Information
