# Supplementary material for: Development of an SNP-based high-density linkage map and QTL analysis for bruchid (Callosobruchus maculatus F.) resistance in black gram (Vigna mungo (L.) Hepper)
Source: Sci Rep. 2019 Mar 8;9:3930. doi: 10.1038/s41598-019-40669-5 (PMC6408486; doi:10.1038/s41598-019-40669-5)
Supplement: Supplementary file 1 — Supplementary Information [file 41598_2019_40669_MOESM1_ESM.pdf]

Supplementary Information

Development of an SNP-based high-density linkage map and QTL analysis for bruchid (*Callosobruchus maculatus* F.) resistance in black gram (*Vigna mungo* (L.) Hepper)

Prakit Somta[1,2,3]*, Jingbin Chen[1], Chutintorn Yundaeng[1], Xingxing Yuan[1], Tarika Yimram[2], Norihiko Tomooka[4], Xin Chen[1]*

[1] Institute of Industrial Crops, Jiangsu Academy of Agricultural Sciences, 50 Zhongling Street, Nanjing 210014, China

[2] Department of Agronomy, Faculty of Agriculture at Kamphaeng Saen, Kasetsart University, Kamphaeng Saen Campus, Nakhon Pathom 73140, Thailand

[3] Center for Agricultural Biotechnology (AG-BIO/PEDRO-CHE), Kasetsart University, Kamphaeng Saen Campus, Nakhon Pathom 73140, Thailand

[4] Genetic Resources Center, Gene Bank, National Agriculture and Food Research Organization, 2-1-2 Kannondai, Tsukuba, Ibaraki 305-8602, Japan

*** Correspondence:** Dr. Prakit Somta, Department of Agronomy, Faculty of Agriculture at Kamphaeng Saen, Kasetsart University, Kamphaeng Saen Campus, Nakhon Pathom 73140, Thailand. Email: agrpks@ku.ac.th, Dr. Xin Chen, Institute of Industrial Crops, Jiangsu Academy of Agricultural Sciences, 50 Zhongling Street, Nanjing 210014, China. Email: cx@jaas.ac.cn

Supplementary Table S1. Locations of major QTLs for *Callosobruchus maculatus* identified in different *Vigna* species on reference genome of mungbean (*Vigna radiata*) and azuki bean (*Vigna angularis*)

***Vigna* species**	**QTL name**	**Linkage group (s) (mungbean/azuki bean chromsome)**	**Reference**
Vigna mungo	*qVmunBr6.1* and *qVmunBr6.1*	6 (Mungbean chromosome 8; azuki bean chromosome 8)	This study
Vigna radiata	*qBr*	2 (Mungbean chromosome 5; azuki bean chromosome 2)	Chotechung et al. (2016)
Vigna umbellata	*Brm3.2*	1 (Mungbean chromosome 7; azuki bean chromosome 1)	Somta et al. (2006)
Vigna nepalensis	*Brm1.3.1*	3 (Mungbean chromosome 7; azuki bean chromosome 3)	Somta et al. (2008)

Supplementary Table S2. Locations of markers associated with black gram QTLs *qVmunBr6.1* and *qVmunBr6.2* for *Callosobruchus maculatus* resistance on reference genome of mungbean, azuki bean and cowpea. Markers associated with *qVmunBr6.1* and *qVmunBr6.2* are highlighted in yellow and green, respectively. Only partial linkage is shown.

LG	Marker name	Position (cM)	Location on mungbean genome	Location on azuki bean genome	Location on cowpea genome
6	Marker3919	6.5	Chr08:42073752	Chr08:3988657	Chr07:37052059
6	Marker14537	7.7	Chr08:42384460	Chr08:3652889	Chr07:37399502
6	Marker14536	8.3	Chr08:42384460	Chr08:3652889	Chr07:37399737
6	Marker14535	8.9	Chr08:42384460	Chr08:3652889	Chr07:37399737
6	Marker12538	9.5	Chr08:42355695	Chr08:3684510	Chr07:37361130
6	Marker30824	10.1	Chr08:42305187	Chr08:3742736	Chr07:37307563
6	Marker21560	10.4	Chr08:42284834	Chr08:3765957	Chr07:37282831
6	Marker19225	10.9	Chr08:42391216	Chr08:3643410	Chr07:37405247
6	Marker19226	10.9	Chr08:42391216	Chr08:3643410	Chr07:37405234
6	Marker14717	11.8	Chr08:41684556	Chr08:4418866	Chr07:36617741
6	Marker12165	12.9	Scafold_665:10479	Chr08:2913663	Chr11:16334603
6	Marker12166	12.9	Chr06:8659146	Chr10:2913663	Chr11:16334603
6	Marker4344	16.3	Chr07:2364670	Chr10:17351119	Chr11:5987397
6	Marker4343	17.2	Scafold_227:266457	Chr09:18556815	Chr03:47538364
6	Marker2358	19.6	Chr08:38524187	Chr09:25927724	No hit
6	Marker17781	20.6	Chr08:40880331	Chr08:6215663	Chr07:35032102
6	Marker28338	21.9	Chr08:40431224	Chr08:6710007	Chr07:34551165
6	Marker14881	22.1	Chr08:39938690	Chr08:7273693	Chr07:34054059
6	Marker9422	22.9	No hit	No hit	No hit
6	Marker9514	24.0	Chr08:3993767	Chr08:7274445	Chr07:34053334
6	Marker15884	26.0	Chr08:40097725	Chr08:7100761	Chr07:34204877; Chr07:34191111
6	Marker13705	28.4	Chr08:37298728	Chr08:9688364	Chr07:31727405
6	Marker5044	29.5	Chr08:38245241	No hit	Chr07:32780313
6	Marker16611	30.3	Chr08:38142865	Chr08:8745683	Chr09:4243826
6	Marker16612	30.4	Chr08:38142865	Chr08:8745683	Chr09:4243826
6	Marker16613	30.5	Chr08:38142865	Chr08:8745683	Chr09:4243826
6	Marker11662	32.2	Chr08:38142872	Chr08:8745676	Chr07:32652762
6	Marker19981	33.1	Chr08:38121671	Chr08:8765777	Chr07:32635561
